# Influence of Temperature on Age-Stage, Two-Sex Life Tables for a Minnesota-Acclimated Population of the Brown Marmorated Stink Bug (*Halyomorpha halys*)

**DOI:** 10.3390/insects11020108

**Published:** 2020-02-07

**Authors:** Byju N. Govindan, William D. Hutchison

**Affiliations:** Department of Entomology, University of Minnesota, St. Paul, MN 55108, USA; hutch002@umn.edu

**Keywords:** brown marmorated stink bug, temperature, development, intrinsic rate of increase, thermal optimum, age-stage two-sex life tables

## Abstract

Temperature is a critical single factor influencing insect population dynamics, and is foundational for improving our understanding of the phenology of invasive species adapting to new agroecosystems or in the process of range expansion. An age-stage, two-sex life table was therefore developed to analyze fundamental demographic features such as development, survival, and reproduction of a Minnesota-acclimated population of the invasive brown marmorated stink bug (*Halyomorpha halys*), in the north central USA. All salient life history parameters were estimated to better understand the population growth potential of *H. halys* at the current limit of its northern range in North America. We examined the effect of selected constant temperatures on immature development and survival (15–39 °C), adult reproduction and longevity (17–36 °C) of H. halys in the laboratory. The Minnesota population developed faster and survived at higher rates relative to a population that had previously established in Pennsylvania, USA. Mean generation time for the Minnesota population was minimized at 30 °C, while survival and fecundity were maximized at 27 and 23 °C, respectively. Given these findings, we assessed the effect of temperature on the intrinsic rate of increase ($r_{m}$), the life table parameter that integrates the effects of temperature on development, survival, and reproduction. A Ratkowsky model predicted $r_{m}$ was maximized (0.0899) at 27.5 °C. We discuss the implications of our findings for understanding population growth rates for *H. halys* in the context of a warming climate, and potential to emerge as a serious crop pest in the Midwest U.S. region.

## 1. Introduction

The development and use of life tables to enhance our understanding of arthropod population dynamics continues to be an informative approach [1]. During the past decade, there have been several improvements in the development of age- and stage-specific life table methodology. Specifically, life tables integrating both age-stage and two-sex components have proven to be most appropriate when estimating life history parameters for age- and stage-structured organisms, including insect species [2,3]. The age-stage, two-sex life table analysis utilizes a sequential schedule of daily (or frequent) survival for both sexes and female fecundity data in the life-cycle of a species. The analysis allows for estimating important life history traits such as age-specific survival, age-specific fecundity and generation time, typically in response to constant or fluctuating temperatures, or a variety of environmental conditions [2,4]. Ultimately, the goal of life table analysis is to provide estimates of salient demographic parameters such as net reproductive rate ($R_{0}$), intrinsic rate of increase ($r_{m}$), finite rate of increase (*λ*), and mean generation time (GT). Traditional age-specific life tables based on females only [5,6] ultimately skew the actual life history curves [1,7]. In contrast, the age-stage, two-sex life table integrates the effect of sex ratio, stage differentiation and variability in developmental rates among sexes; this approach thus provides more accurate estimates of population parameters [3]. Age-stage, two-sex life tables are therefore a valuable tool for analyzing the life history strategies of insect species, particularly in response to constant or fluctuating temperatures, as this variable is known to be among the most critical to insect population dynamics [2,8,9,10,11].

The brown marmorated stink bug, *Halyomorpha halys* (Stål) (Hemiptera: Pentatomidae), native to Northeast Asia, primarily China, Korea and Japan [12], has rapidly spread globally in the past 10–15 years [13,14], including much of the U.S., and western and eastern Europe. Specifically, *H. halys* is predicted to potentially have high risk of invasion into climatically suitable regions between 30° and 50° latitude in both the northern and southern hemispheres [12,15], and the species continues to expand its global distribution outside its native region as a polyphagous pest of more than 170 crops and non-crop plants [13,16,17]. In North America, established populations of the pest have been reported from 44 contiguous states of the US and four Canadian provinces [18]. In South America, *H. halys* has been detected as far south as Chile [19]. Throughout Eurasia, the pest is now established in Switzerland [20], Liechtenstein [21], Germany [22], France [23], Italy [24], Greece [25], Hungary [26], Romania [27], Serbia [28], Republic of Georgia [29], Spain [30], Russia and Abkhazia [31,32], Bulgaria [33], Slovakia [34], Kazakhstan [35], with latest intrusions reported from Croatia [36], Turkey [37], and Malta [38]. In North America *H. halys* was first officially reported in 1996, from Allentown, Pennsylvania [39]. Within a decade, *H. halys* emerged as a serious invasive pest of crops in the eastern United States, resulting in ~$37 million (USD) in losses to the Mid-Atlantic apple industry [40], and 100% losses to peaches in Maryland [13] in 2010. In Minnesota, *H. halys* was first detected on 18 November 2010 [41] and is currently a nuisance home invader, as well as an emerging threat to agriculture [42,43]. Reproducing populations that consistently overwinter have been detected since 2011 in a residential area of Wyoming, MN, just north of St. Paul [42]. The immatures have also been detected in soybean fields since 2017 [43], suggesting the presence of breeding populations. If the invasion of *H. halys* in Minnesota and the Midwest progresses as it has in other states, significant infestations of the pest in agricultural crops are likely to occur in the near future; initial crops at risk include soybean, sweet corn, apples, and grapes. Our proactive research was initiated to better understand the population ecology and biotic potential of this invasive species during the early establishment phase in the Minnesota.

A thorough understanding of the arthropod pest demography, particularly for invasive species, in relation to ambient temperature is crucial to formulate population growth models [44], and ultimately facilitate ecologically based integrated pest management programs (IPM) in novel environments. As with most insects, the invasion, establishment and range expansion of *H. halys* in the US is primarily influenced by temperature and its interplay with photoperiod [10]. Previous research has assessed the effect of diurnally varying temperature on the developmental and reproductive phenology of a northern Italian [44] and Swiss [45] populations of *H. halys* under field conditions. The influence of temperature and/or photoperiod on the developmental and/or reproductive phenology of *H. halys* adapted to different eco-climatic regions of the world have been studied, including a Pennsylvania (Allentown, PA, USA), Oregon (OR, USA) and Korean population, in controlled lab settings [10,45,46,47,48] and applied to develop simulation models of the pest [49]. For the Pennsylvania study, the primary focus was estimating the impact of constant temperatures on development time on an eastern US population of *H. halys*, and age-specific fecundity was measured only at 25 °C [47]. By contrast, the Oregon study primarily focused on the effect of temperature on ovipositional and adult survival thresholds of a Northwestern US population of *H. halys* and ignored egg-to-adult development time and survival [48]. Accordingly, Maslen in Oregon [48] and Baek et al. in Korea [46] demonstrated the temperature dependency of fecundity for *H. halys*. Further, Nielsen et al. [47] and Maslen [48] also estimated three life history parameters—$R_{0},\;T$ and $r_{m}$—for *H. halys*, but relied on the conventional female-only life table approach and were based on a limited number of temperatures. For example, life history parameters for the PA population were estimated for only 25°C and were adjusted to account for the number of first instars emerging [47]. Also, life history parameters for the OR population in Northwestern US [48] were estimated using the egg-to-adult survival data for the genetically distinct PA population in the eastern US [47,50], and fecundity data for the OR population [48]. Therefore, the reliability of Maslen’s estimate of life history parameters for the OR population at different constant temperatures [48] is problematic. To our knowledge, the effect of multiple constant temperatures on the development and reproductive phenology for any population of *H. halys* in the laboratory has been limited to the Korean population [46]. Life history parameters for the genetically distinct Korean population [50] are not fully informative to model the phenology and population dynamics of a genetically distinct population of *H. halys* in the US or elsewhere. Temperature-dependent life history parameters developed for a pest species can vary by eco-climatic region [6], and subsequent models can only be as informative as the data driving these models.

Clearly, comprehensive knowledge on the life history parameters of the MN acclimated population and in particular the Midwest US populations of *H. halys* at a range of constant thermal conditions is needed for improving our understanding of this species, and develop simple day-degree models or temperature-driven phenology and population dynamics models to inform integrated management of this pest. Therefore, the aim of our study was to employ an age-stage, two-sex life table approach to assess the effect of different constant temperatures on life history parameters of a Minnesota population (hereafter referred to as MN) of *H. halys* under controlled laboratory conditions in the absence of natural enemies. The information gained will help the researcher community better appreciate the biological potential of the pest in its novel natural environment in Minnesota, which currently represents the northern limit of its range in North America. Additional knowledge will also provide novel insights on the biology, developmental, reproductive, and generational dynamics for this pest in the Midwest US region.

## 2. Material and Methods

### 2.1. Laboratory Colony

The laboratory colony of *H. halys* was initiated in 2012 [42,51], with adults collected from a home in a residential development, Wyoming, Minnesota (45.33N-92.99W) [42]. *H. halys* has been reported as a nuisance pest at this location since this time, and soon after the first detection in Minnesota in 2010 [41]. We have observed *H. halys* at this location since 2012, where both adults and nymphs have been collected on *H. halys* pheromone traps [52], which is indicative of a locally reproducing population. For our study, 80–100 adults were collected each year from this location in September–October, and subjected to diapause conditions in the lab; surviving adults were then added to our *H. halys* colony to maintain genetic diversity (October, 2015, 2016). To establish non-diapausing *H. halys* colonies for our study, 20–25 adults from the main laboratory colony were reared in two large ventilated plastic containers. Each cage was provisioned with paper towels as substrate for overwintering and held in the environmental chamber (Percival Scientific, Inc., Perry, IA, USA) at 10 ± 1 °C and under short-day conditions (8L: 16D) with relative humidity of 40–60%. Starting in January-February of each year, overwintered adults were added to adult rearing cages (60.96 × 60.96 × 142.24 cm; BioQuip, Catalog #1466DV), held in walk-in-chambers at 24 ± 1 °C, relative humidity (RH) of at least 65% and a 16L: 8D photoperiod to break diapause and maintain a reproducing lab colony. The spring-summer rearing protocol of Iverson et al. [51] was used to minimize cannibalism by 2^nd^ instars. Specifically, we provisioned each cage with flats containing 10–12 common bean seedlings as the substrate for mating and oviposition and replaced the flats as needed. Organic food, particularly fresh green beans (*Phaseolus vulgaris* L.), carrot (*Daucus carota* subsp. *Sativus*), seeds of dry soybean (*Glycine max* (L.) Merril) and sunflower (*Helianthus annuus*) purchased from the local market were provided to bugs; the food was replaced at least thrice weekly. Eggs were collected from the adult cages on a daily basis and reared in Petri dishes with moistened filter paper (Fisher Scientific, P5; 9.0 cm diameter, Pittsburgh, PA, USA) to second instar nymphs. The second instars were then transferred to separate nymphal rearing cages maintained under the same conditions and development was monitored on a routine basis. A portion of the emerging adults of the subsequent generation was returned to adult rearing cages to maintain the lab colony. Temperature and RH data in all chambers were recorded using a Hobo data logger (Hobo U23 Pro V2, Onset Computer Corporation, Bourne, MA, USA).

### 2.2. Developmental Time and Life Table Studies

The *H. halys* immature (egg-to-adult) developmental time and survival was monitored in environmental chambers programmed at 10 constant temperatures (15, 17, 20, 23, 25, 27, 30, 33, 36, and 39 °C), 16L: 8D photoperiod and 65–75% RH. Throughout the study, eggs, nymphs and adults were monitored every 12 hours on a daily basis, following a protocol modified after Nielsen et al. [47]. To initiate each life table study, a minimum of 50 fresh egg masses laid (exception: 35 egg masses at 20 °C) within 6 hours were obtained from females held in adult rearing cages at the respective temperatures (except for the 15 and 17 °C, temperatures below the lower threshold for reproductive maturation and egg laying by *H. halys*). The collected eggs were then transferred on to a moistened filter paper placed inside the lid of an inverted sterile polystyrene Petri dish (Fisher Scientific, 100 mm × 20 mm, Pittsburgh, PA, USA). For the treatments at 15 and 17 °C, fresh eggs laid within 6 hours by mated adult females at 20 °C were used. Egg masses and nymphs were held in Petri dishes provisioned with moist filter paper and 25 0.5 mm diameter holes on the lid for ventilation. First instars from each egg mass were allowed to aggregate and develop in the same Petri dish. All individuals that survived to second instar were transferred to individual Petri dishes with the afore-mentioned design to record developmental time and daily survival until adult emergence. Filter paper was moistened as needed, and pieces of fresh organic green beans, baby-cut carrots, and dry soybeans were provisioned as food and replaced every two-three days; the food was never a limiting factor. Stage-specific developmental time and survival were recorded daily by monitoring for the shed exuvia and based on nymphal characteristics following Hoebeke and Carter [39]. Shed exuvia marked the successful completion of a current instar, and exuvia were removed immediately. Freshly emerged adults were sexed to develop the age– and stage-specific life table for each temperature.

For the fecundity study, growth chambers, programmed at seven constant temperatures were used, including: 17, 20, 23, 27, 30, 33, and 36 °C; no adults developed at 15 and 39 °C, and thus fecundity was not monitored at these temperatures. Newly eclosed male and female adults, within a 12 hr period, from the immature development study, were used. One male and female pair were each placed in 946 ml translucent plastic containers (13.97 cm tall × 11.56 cm diameter top × 9.02 cm diameter base). If the number of males emerged was less than the females on a given day in any treatment, then males from an extra set of insects reared in parallel at the respective temperature were used. The lid (10.16 cm dia) and two rectangular cross-sections (7.62 cm length × 3.81 cm width) of the container were replaced with insect screen mesh (*No-See-Um*) for ventilation. A 7.62 cm green floral polypropylene water tube (Royal Imports, Brooklyn, NY) was inserted through about a 1cm diameter hole made at 7.62 cm height from the base of the container. The water tube was then glued at an inclined angle of 330° relative to the base of the container, such that one-fourth of the water tube with its cap end is inside the container and its remaining two-third is protruding out of the container. A slit was made on the center of the cap of the water tube and a single-stem of green beans (Romano Bush #14) cut at a slanting angle below the internode was placed through the stretchable middle opening in the cap of the floral water tube. This set up allowed forming a water-tight seal to prevent the water tube from leaking and inhibited the entry of *H. halys* nymph or adult into the water vial. The plant served a substrate for the *H. halys* to mate and lay eggs, preferably on the underside of the leaves. A one mm diameter circular hole was provisioned on the top part of the water tube extending out of the container; the hole allowed routinely fill water into the tube without the need to open the container and the cap of the water tube, and slowed down the wilting of plants by a couple of days. Plant stems were replaced every two-four days in all replicate containers throughout the study, depending on the treatment temperature. The replicate mating pairs used per treatment varied (3 to 19) depending on the number of individuals that survived to adults (sex ratio was 1:1) in the developmental phenology study. 

For each replicate in all treatments, we recorded daily whether the individual was alive or dead, the female has laid any egg mass and the total number of eggs per egg mass. The egg masses were gently pulled off the leaves and transferred on to a moist filter paper placed inside a Petri dish and labeled by date and replicate ID to monitor for successful hatching. Each replicate was monitored until the death of females and males. When a male died, another male of similar age replaced the dead one. Males were rotated between the replicate containers weekly to reduce any adverse effects on their fitness affecting the female fecundity.

### 2.3. Statistical Analysis

The life history data of *H. halys* collected at different constant treatment temperatures were analyzed following the age-stage two-sex life table theory [2,3]. The analysis was carried out using the TWOSEX-MSChart program [53]. We estimated the mean (± S.E.) developmental time for egg and the five nymphal instars (first to fifth instar), the adult pre-oviposition period (the time period between female adult emergence and its first egg-laying), the oviposition period, the fecundity and the longevity of the males and females in number of days.

For the age-stage, two-sex life table, the population parameters were calculated based on the $S_{xj}$ (the survival rate of *H. halys* in age x and stage j), $f_{xj}$ (the age-stage-specific fecundity defined as the daily number of eggs produced by females at age x and stage j), $l_{x}$ (age-specific survival rate defined as the probability that a newly laid egg survives to age x; see Equation (1)), and *m_x_* (age-specific fecundity defined as the daily number of eggs produced by females at age x; see Equation (2)). They were calculated for each cohort from the daily record of the survival and fecundity of all individuals in the cohort. For *H. halys*, the first stage corresponds to egg, stage 2 to 6 correspond to the first to the fifth instar, stage 7 corresponds to female and stage 8 to male.

(1) $$l_{x}=\;\sum_{j=1}^{k}S_{xj}$$

(2) $$m_{x}=\frac{\sum_{j=1}^{k}S_{xj}f_{xj}}{\sum_{j=1}^{k}S_{xj}}$$

where *k* denotes the number of life stages. The net reproduction rate *R*_0_ (the total number of offspring that an individual can produce during its lifetime; see Equation (3)), the gross reproduction rate GRR (see Equation (4)) and the intrinsic rate of increase $r_{m}$ (the rate of population increase per unit time; see eqn. 5; [3]) were calculated from $l_{x}$ and $m_{x}$ as follows:

(3) $$R_{0}=\;\sum_{x=0}^{\propto }l_{x}m_{x}$$

(4) $$GRR=\sum m_{x}$$

(5) $$\sum_{x=0}^{\propto }e^{-r_{m}\left(x+1\right)}l_{x}m_{x}=1$$

The intrinsic rate of increase ($r_{m}$) is estimated via the iterative bisection method from the Euler-Lotka formula (Equation (5)), with age indexed from 0 [54]. The finite rate of increase λ (the number of females added to the population per female per unit time; see Equation (6)) and the mean generation time T (the length of time needed for a population to increase to $R_{0}$ fold of its size at the stable age distribution; see Equation (7)) [53] are calculated as,

(6) $$\lambda =e^{r}$$

(7) $$T=\frac{\ln R_{0}}{r}$$

The age-stage life expectancy $e_{xj}$ (the time that an individual of age *x* and stage *j* is expected to live; see Equation (8)) [55] and the age-stage reproductive value $v_{xj}$ (contribution of individuals of age *x* and stage *j* to the future population; see Equation (9)) [56] are estimated as:

(8) $$e_{xj}=\sum_{i=x}^{\alpha }\sum_{y=j}^{k}{S^{\prime }}_{iy}$$

(9) $$v_{xj}=\frac{e^{r\left(x+1\right)}}{S_{xj}}\sum_{i=x}^{\alpha }e^{-r\left(i+1\right)}\sum_{y=j}^{k}{S^{\prime }}_{iy}f_{iy}$$

where ${S^{\prime }}_{iy}$ is the probability that individuals of age *x* and stage *j* will survive to age *I* and stage *y*. 

For each life stage, including the pre-adult stage (egg-to-adult emergence), development times at different constant temperatures were compared with one-way analyses of variance (ANOVA), followed by pairwise comparison of means using Tukey HSD test at 5% significance level.

For all life table parameters other than development time, we estimated the means and standard errors using the bootstrap procedure [57] included in the TWOSEX-MS chart software. The bootstrap analysis uses random sampling. With a small number of samples, it will generate variable means and standard errors. Hence, we used 10,000 random resampling in order to reduce the variability of the estimates. The paired bootstrap test was used to test for significant differences in adult pre-oviposition period, oviposition period, fecundity, adult longevity, and other derived parameters between the treatment temperatures at 5% level of significance [7,57]. Mean developmental time for different stages of the MN population of *H. halys* at 15, 17, 20, 25, 27, 30 and 33 °C were also compared with the corresponding published estimates for the PA population of *H. halys* [47]. Independent two-sample Student’s t-test or Welch’s t-test, depending on whether sample variances are equal or unequal, respectively, was used for the comparison of mean development times.

We also modeled the effect of temperature on intrinsic rate of increase $\left(r_{m}\right)$ estimated via age-stage two-sex life table analysis. Currently there exists no extensions of reaction rate theory that incorporate the kinetics of several major enzymes that each drive distinct fitness components such as survival, reproduction and development [58], but empirical and biophysical models describing the effect of temperature on developmental rate have been used previously to describe the relationship between temperature and $r_{m}$ [59,60] in insect species. Accordingly, we fitted all the models included in *devRate* package [61] and the thermodynamic Sharpe–Schoolfield–Ikemoto model of the OPTIMSSI package [62] in R freeware [63], to explain the temperature dependency of $r_{m}$. The model with lowest value for Akaike Information Criterion (AIC) was chosen as the best fit model [64]. The temperature corresponding to maximum point of the fitted regression curve for the best model represented the optimal temperature for $r_{m}$.

## 3. Results

### 3.1. Developmental Time, Adult Longevity and Lifespan

The mean developmental duration of the egg stage, the five instars, and total pre-adult stages of the MN-acclimated *H. halys,* at constant temperatures between 15 and 36 °C, as well the pre-adult survival rates at corresponding temperatures are summarized in Table 1. Complete development of any given cohort from egg to adult occurred only from 17 to 36 °C. The temperature had a significant effect on the pre-adult development time of *H. halys* (*F_7, 196_* = 476.23, *p* < 0.001), and non-linear trends were evident with rise in temperature (Table 1). Pre-adult developmental time was highest at 17 °C (103.89 ± 4.69 days) and the shortest at 30 °C (30.95 ± 0.45 days). Pre-adult developmental times at 17, 20, 23 and 25 °C were also significantly different from each other (*p* < 0.001) and estimates at temperatures ≥27 °C (*p* < 0.001), but there were no significant differences in development time (*p* > 0.05) for 27, 30, 33 and 36 °C. For all temperatures tested, pre-adult developmental times were not significantly different for males and females, and the sex ratio at adult emergence was nearly 1:1.

Temperature significantly affected the stage specific mean development times also of *H. halys* (Table 1). As expected, temperature significantly affected the development time of egg (*F_8, 516_* = 13204.86, *p* < 0.001), first (*F_8, 400_* = 6043.81, *p* < 0.001), second (*F_7, 367_* = 537.57, *p* < 0.001), third (*F_7, 282_* = 108.61, *p* < 0.001), fourth (*F_7, 238_* = 76.75, *p* < 0.001) and fifth instars (F_7, 198_ = 84.06, *p* < 0.001). The point at which development rate (inverse of developmental time) began to decline for the second and fifth instar nymphs, however, was recorded at 33 and 27 °C, respectively. At 15 °C, the eggs and first instar nymphs completed development, but experienced 98% mortality as second instars, with only a few molting to the third instar. Interestingly, however, when newly emerged healthy second instars reared at 27 °C were transferred immediately to 15 °C, they completed development to adults at 15 °C. The developmental time (mean ± standard error) for the corresponding second, third, fourth, and fifth instar stages of *H. halys* at 15 °C were 38.25 ± 1.37, 32.59 ± 1.04, 28.59 ± 1.23, and 43.73 ± 1.72 days respectively. Thus, if the MN population of *H. halys* adapts in the future to complete egg-to-adult development at 15 °C, the corresponding pre-adult developmental time would approximate to 186.23 ± 0.78 days. At the highest temperature tested (39 °C), developmental time could not be measured as egg mortality was 100%. 

The developmental times at 17, 20 and 23 °C were significantly different (*p* < 0.001) for each life stage (egg, first, second, third, and fourth instar) except the fifth instar (Table 1). Mean developmental times for the fifth instar at 17and 20 °C differed significantly (*p* < 0.001) from that at 23 °C, but not from each other. Between 23 and 25 °C, developmental times were not significantly different for any life stage except for the second instar (*p* < 0.001) and fifth instar (*p* < 0.001). For temperatures between 27, 30, 33, and 36 °C, no significant difference in developmental time was evident for the fourth as well as the fifth instar. Similar trends were evident for the most part for all other life stages at these temperatures (27–36°C), except for the second instar. The second instar was the only life stage with significantly different developmental times (*p* < 0.05) recorded at all temperatures except between 30 and 36 °C.

Adult longevity did not differ between two sexes, was highest at the extreme low temperature (17 °C) (Table 2) and lowest at the extreme high temperature (36 °C). Female and male adult longevity (in days) at 36 °C were 19.60 ± 2.75 and 15.37 ± 1.73, respectively. There were significant differences (*p* < 0.05) in the longevity of females at 20, 23 and 27 °C. In addition, male longevity at 30 °C was also significantly different (*p* < 0.05) from that at 20, 23, and 27 °C.

### 3.2. Pre-Oviposition Period, Oviposition Period and Fecundity

The adult pre-oviposition period was longest at 20 °C (32.67 ± 1.37 days) followed by that at 23 and 27 °C, where the mean times were significantly different (Table 2, *p* < 0.05). The pre-oviposition period was the shortest at 27, 30, and 33 °C, and ranged from 10.84 to 11.54 days, but were not statistically different (Table 2, *p >* 0.05, paired bootstrap test). At 17 °C, females of *H. halys* did not produce any eggs. Among all treatments, the oviposition period differed only between 20 and 30 °C (Table 2, *p* = 0.002); the oviposition period also recorded numerically the highest at 20 °C (70.13 ± 12.33 days). Fecundity was numerically highest at 23 °C (308.42 ± 30.24 eggs per female; Table 2). Fecundity at 23 °C as well as 27 °C was significantly higher (*p* < 0.05) than that at 30 and 33 °C only; otherwise, no differences were detected. At 36 °C, all 20 mating pairs died within six weeks of emergence, while the adult pre-oviposition period increased to 16.67 ± 1.67 and mean fecundity was negligible (1.85 ± 1.17).

### 3.3. Life Table Analysis

**Figure 1:**
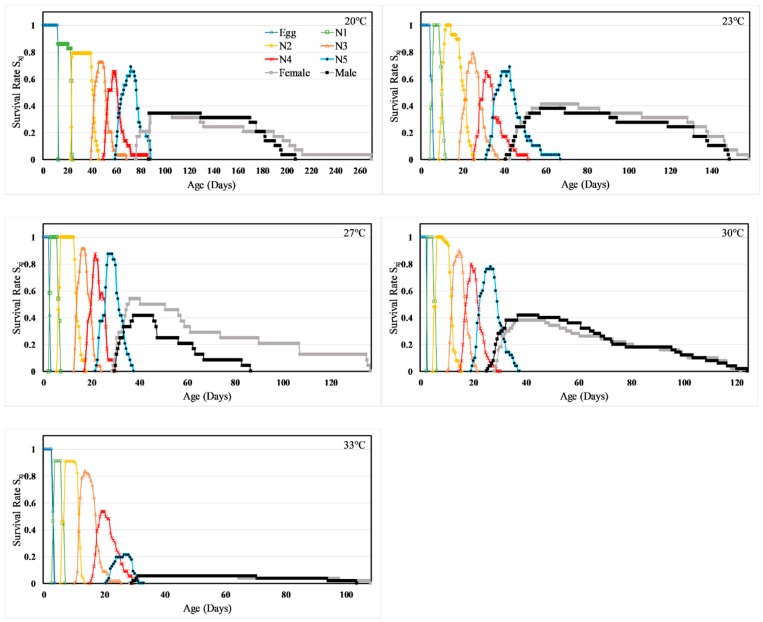
Temperature dependent age-stage-specific survival rate (*S_xj_*) of *H. halys.*

The cohort-specific egg-to-adult survival rates increased gradually as temperature increased above 15 °C, peaked at 27 °C (96%) and declined thereafter (Table 1). Owing to the variable developmental rates among individuals, there was a significant overlap in age-stage specific survival rates (*S_xj_*) (Figure 1; also see, Supplementary Table S1). Egg-to-adult survival rates were not statistically different (*p* > 0.05) at 15 °C (0%) and 36 °C (2%), at 17 °C (17%) and 33 °C (11%), as well as at 23, 25 and 27 °C. Stage-specific survival rates for the egg and instars 1–2 were lowest at the extreme low (15 °C) and the extreme high temperatures (36 °C), and peaked to near 100% at intermediate temperatures between 20 and 30 °C. For late instars, survival was very high at low to intermediate temperatures (20–30 °C), but declined abruptly to experience the highest mortality at the extreme temperatures of 33–36 °C.

**Figure 2:**
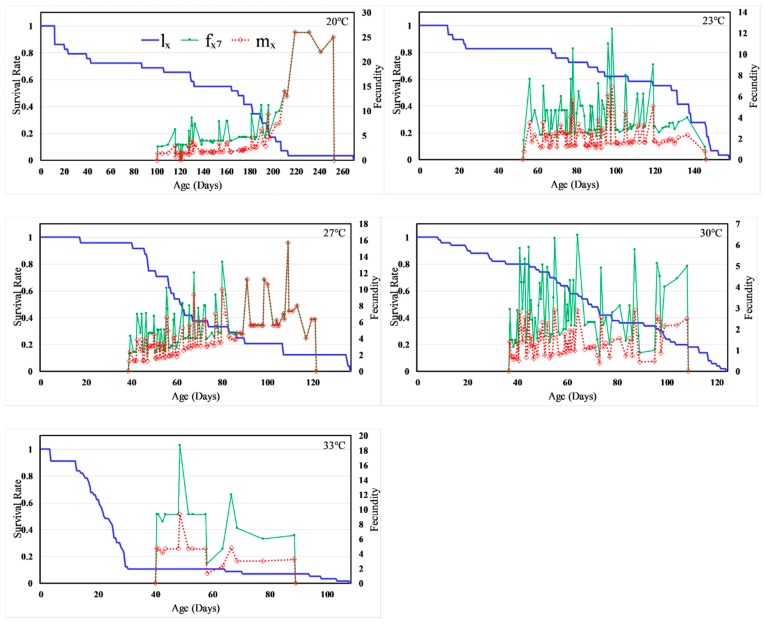
Temperature dependent age-specific survival rate (lx), female age-stage-specific fecundity ($f_{x7}$), and age-specific fecundity (mx) of *H. halys*.

Age-specific survival rate (*l_x_*) ignoring the stage differentiation (probability that an egg will survive to age x) and the age-stage specific fecundity (the number of eggs produced by adult females of age *x* where the age *x* is counted from the egg stage) and age-specific fecundity (*m_x_*) are plotted in Figure 2. The *m_x_* curve suggests that the first reproduction began at day 100, 52, 38, 36 and 40 at 20, 23, 27, 30 and 33 °C, respectively, with the corresponding oviposition period spanning 152, 94, 83, 72 and 49 days, respectively. The *l_x_* survivorship curve for the population at all but 33 °C, in general, reveals a very low but equally probable rate of mortality for all life stages, representing a type II diagonal survivorship typical for the insects [1]; the mortality increased disproportionately for the late instars relative to early stages at 33 °C. The values of *l_x_* at first reproduction were 69%, 83%, 96%, 80% and 11% at 20, 23, 27, 30, and 33 °C respectively; these estimates correspond relatively well with the pre-adult survival rates. Also, Figure 2 illustrates temperature-dependent reproduction of *H. halys* as an interplay among $f_{x7}$ (females represent stage 7 in the life cycle and *x* denotes age), *l_x_* and oviposition duration, and indicate that reproductive potential was the highest between 23 and 27 °C.

Paired bootstrap analysis revealed significant differences in life history parameters—mean generation time (T), net reproduction rate (R_0_), gross reproduction rate (GRR), intrinsic rate of increase ($r_{m}$) and finite rate of increase (λ)–of *H. halys* estimated at different treatment temperatures (Table 3, *p* <0.05). The mean generation time at 20, 23 and 27 °C was significantly different (*p* < 0.05, paired bootstrap test), was highest at 20 °C (145.60 ± 5.89), lowest at 30 °C (50.94 ± 1.55) and recorded a numerical increase at 33 °C (Table 3). The R_0_ increased with temperature and peaked at 27 °C (151.06 ± 41.16) but was not significantly different (*p* < 0.05) from that at 20 and 23 °C (Table 3). The R_0_ dipped at 30 °C (58.12 ± 12.02), and further at 33 °C (6.80 ± 3.75) which was significantly different from R_0_ at all other treatment temperatures. The GRR recorded the highest at 27 °C (401.65 ± 55.57 offspring/individual; Table 3), while the lowest was at 33 °C (66.81 ± 31.86) with the trend in turn being rates at 27 > 23 > 30 > 33 °C. The GRR at 20 °C (229.4 ± 84.03) was not significantly different from any of the treatment temperatures. The $r_{m}$ (0.0876 ± 0.00 day^−1^) and λ (1.092 ± 0.00 day^−1^) were the highest at 27 °C and they were significantly higher than all corresponding treatment estimates but that at 30 °C (Table 3). The $r_{m}$ (0.0286 ± 0.00 day^−1^) and λ (1.029 ± 0.00 day^−1^) were found to be the lowest at 20 °C, but not significantly different from corresponding estimates at 33 °C.

The temperature had a non-linear effect on $r_{m}$. The Ratkowsky model $\left[r_{m}\left(T\right)∼{\left(cc*\left(T-T1\right)*\left(1-\exp\left(k*\left(T-T2\right)\right)\right)\right)}^{2}\right]$, with the lowest AIC (−59.52; See Supplementary Table S2) among all non-linear models considered, best described the relationship between temperature and $r_{m}$ for *H. halys*. Ratkowsky model predicted the lower (T1) and higher (T2) temperature thresholds for $r_{m}$ as 15.66 and 35.96 °C, respectively, although we observed no egg-laying at 17 °C. The coefficients cc and k were respectively, estimated to be 0.05 and 0.07. The $r_{m}$ was maximized (0.0899 day^−1^) at 27.49 °C.

**Figure 3:**
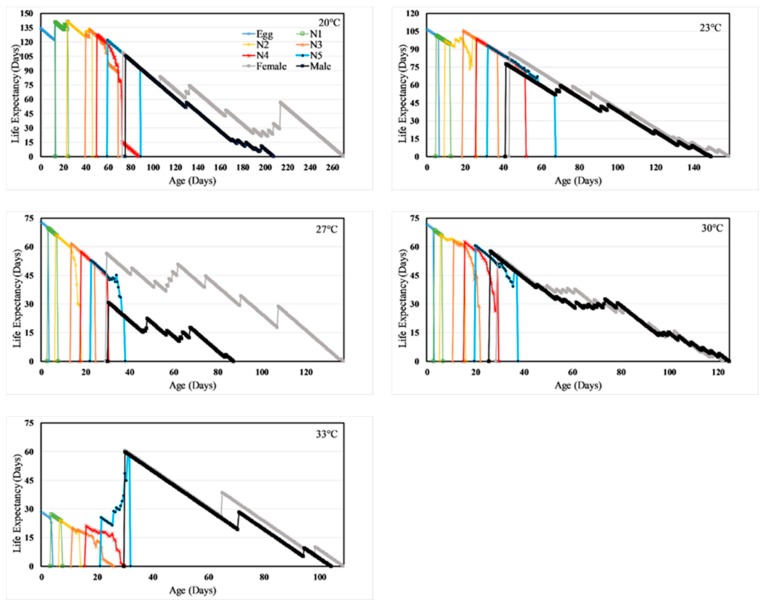
Temperature dependent age-stage-specific life expectancy (*e_xj_*) of *H. halys.*

**Figure 4:**
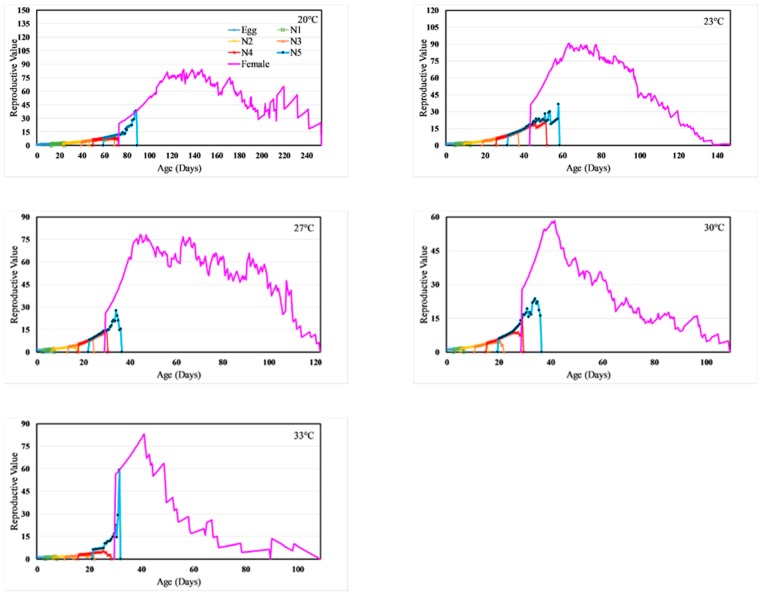
Temperature dependent age-stage-specific reproductive value (*v_xj_*) for *H. halys.*

Age-stage, two-sex life tables also provided estimates of the age-stage specific life expectancy (*e_xj_*; Figure 3) and the reproductive value (*v_xj_*, Figure 4). Explicitly, the life expectancy of a newly eclosed individual at 20, 23, 27, 30, and 33 °C is 134, 106, 73, 72 and 28 days, respectively (Figure 3). A female of age 76 days at 20 °C and age 38 days at 27 °C will be able to live for another three months, while a female of age 38 days at 33 °C will live only for about a month, respectively (Figure 3). The reproductive value for a new egg (*v01*) is the finite rate of increase (*λ*) (Figure 4 and Table 3). For instance, the reproductive value for a new egg at 27 °C is 1.092. The peak reproduction occurred at 106–181, 52–97, 39–80, 35–45, and 32–48 days at 20, 23, 27, 30, and 33 °C respectively, and these days represented the age at which females in respective treatment contributed the maximum to future population.

### 3.4. Comparison of Development and Survival Rates for the MN versus PA populations of H. halys

At all comparable temperatures, except for 20 °C, the MN-acclimated population of *H. halys* developed faster and exhibited higher survival rates than the PA population [47] (See Supplementary Table S3). For the MN population, the development of the pre-adult stage at all but 20 °C (*p* <0.05), the eggs at 15, 17, 20, 25, and 27 °C (*p* < 0.001 for all), the first instars at 17, 27 and 30 °C (*p* <<< 0.001), the second instars at 17 °C (*p* = 0.006), 30 °C (*p* = 0.05) and 33 °C (*p* = 6.77 × 10^−10^), third instars at 30 °C (*p* = 0.03) and 33 °C (*p* = 0.02), the fourth (except at 17 and 33 °C) and the fifth instars (except at 27 and 30 °C) was significantly faster than their PA counterparts. By contrast, the first instars of the MN population developed slower at 20 °C (*p* = 2.27 × 10^−18^) and 33 °C (*p* = 4.51 × 10^−6^); this was also true for the second instars at 20 °C (*p* = 4.95 × 10^−7^; Supplementary Table S3). Importantly, all five nymphal stages of the MN population were able to complete stage-specific development at 15 °C; however, indeed 98% stage-specific mortality was evident for the MN second instars. At 36 °C, 2% of the MN population of *H. halys* completed egg-to-adult development, but the eggs of the PA population failed to hatch at 35 °C. Numerically, survival rates at all comparable temperatures tested were higher for the MN vs PA populations; however, statistical comparisons were not possible due to a lack of reported standard errors for the PA population (for estimates of the MN population, See Table 3).

## 4. Discussion

*Halyomorpha halys* has emerged as a serious invasive pest of vegetable, fruit, and ornamental crops in numerous geographic regions of the world and poses a severe threat to Minnesota and Midwestern US agriculture [65]. Models integrating temperature-dependent life history parameters are essential to developing realistic population models and provide real-time forecasts to design sustainable strategies for managing invasive pests like *H. halys* and projecting their dynamics in response to climate change scenarios. The age-stage, two-sex life tables offer an improvement over traditional female-only life tables to improve the resolution of the survival-fecundity schedules and derived life history parameters [2]. The current work is the first study to apply age-stage, two-sex life tables to estimate life history parameters at varying constant temperatures of a *H. halys* population in the Midwest U.S., or elsewhere. Methodologically, our estimates are an improvement over the previous estimates of life history parameters based on traditional female-only life table approach, computed after adjusting for survival rates [7,46,48]. 

Our study is unique in being the first comprehensive laboratory study to investigate and describe the influence of constant temperatures on development, survival, and reproductive rates for a US population of *H. halys* using the two-sex life table approach. Two major studies that assessed the temperature-dependency of US populations of *H. halys* primarily focused on development time [47] or reproductive rates [48]. The only other comparable study for a Korean population [46] did not explore beyond fecundity analysis to estimate and assess temperature dependency of life history parameters. Our findings suggest shorter pre-adult and stage-specific development times, and higher survival rates for the MN population relative to previous estimates for any US population [47,48]. The MN-acclimated population of *H. halys* also had uniquely high fecundity and a shorter pre-oviposition period relative to the Northwestern US populations and Korean populations, the only two other populations for which similar comprehensive study is known to have undertaken to assess the temperature-dependency of fecundity. Most importantly, our data provide the limiting effect of temperature extremes on development, survival, fecundity, and adult longevity for an MN-acclimated population of *H. halys.* It will prove to be informative in developing predictive phenology and population dynamics models for this invasive pest over space and time (e.g., different seasons within a year and also across years), in the Midwest region. Models informed by data from MN-acclimated pest population will be useful in projecting the potential areas of range expansion and the probability of outbreaks in the agro-ecosystems of the Minnesota and Midwest.

Results for the MN-acclimated population of *H. halys* corroborates the non-linear response of developmental time as a function of temperature reported for most insect species [60,66], including stink bug species [67,68] and specifically the PA population of *H. halys* [47]. Pre-adult developmental time decreased steeply with a rise in temperature from 17 °C and then gradually declined as the temperature approached 30 °C, with significant differences in the developmental time among all temperatures from 17 to 27 °C (Table 1). For the pre-adult phase and all life stages of the MN population, the development time was always the longest at 17 °C and shortest at 30 °C; the only exceptions were the shortest development times for the second and fifth instars at 33 and 27 °C respectively (Table 1). Nearly consistent findings were reported for the PA population with egg, first, and second instars at 30 °C and third, fourth and fifth instars at 27 °C, for the shortest development times, respectively [47]. At temperatures below the lower thresholds, the reduced enzyme activity results in slower or negligibly low metabolic processes, which require substantial energy to repair injuries in insects, resulting in longer developmental times [69,70]. The high temperature thresholds of 36.5 and 35.76 °C were previously reported for the Swiss [44] and US (Pennsylvania; [47]) populations of *H. halys*. These estimates are based on models fit to the pest’s phenology data but do not support *H. halys* egg-to-adult development below 17 °C or above 33 °C. In contrast, we provide empirical evidence at constant temperatures where a MN population of *H. halys* exhibited lower and upper developmental thresholds of 17 and 36 °C, respectively. We confirmed that the MN population could complete development at a high thermal threshold of 36°C, despite experiencing near 98% mortality. Our data also show that the ability of the pest to complete egg-to-adult development at 15 °C is primarily constrained by the near 100% mortality in the second instar. As temperatures increase beyond a high threshold, the enzymes needed for metabolic and physiological processes are denatured, leading to an arrest of the developmental events [71]. The long-term exposure of different life stages of insects to either thermal extremes also tend to reflect on their life history as low egg hatch or larval survival to adult [70] or may result in deformed adults.

Pre-adult survival rates of *H. halys* were significantly lower at the lower (15–17 °C) and higher thermal extremes (33–36 °C), with survival rates peaking (96%) at 27 °C (Table 1, Supplementary Table S1). Compared to the PA population [47], the MN population had a relatively higher survivorship of 80–96% between 23 and 30 °C (Table 1), suggesting that this range of temperatures was least stressful on their growth, development, and reproduction. *H. halys* reared at analogous laboratory conditions (mean ± standard error (s. e.) of 27 ± 2 °C; 16:8 L:D and 70 ± 10% RH) on multiple food substrates have been shown to attain >85% egg-to-adult survival rates [72]. However, abiotic factors like lower relative humidity may also constrain stink bug populations from attaining higher survival rates in the field, similar to what has been reported in controlled laboratory conditions [67,73].

Findings from our study on the MN population filled the gap in comprehensive knowledge on both temperature-dependent adult longevity and reproductive parameters for any population of *H. halys* (Table 2) [46,48]. Our estimates of longevity are higher than the only comparable information in the literature for the OR population [48]. The adult pre-oviposition period for the PA population at 25 °C (13.35 ± 0.72) was not different from that for the MN population at 23 and 27 °C (Table 2). While the OR population lack reported standard errors for comparison, the pre-oviposition period for the Korean population (mean ± s. e. of 21.5 ± 2.19 at 25 °C, 14.5 ± 0.85 at 30.3 °C, 15.0 ± 1.20 at 32.7 °C) was considerably longer (*p* < 0.05) [46]. Thermal limits of oviposition for the OR and Korean populations are reportedly 18–33 °C [48] and 18.8–32.7 °C [46], respectively. Thus, while the lower thermal limit of oviposition for MN population could be safely assumed as 18–19 °C based on our findings, the corresponding upper thermal limit of 36 °C adds new data to the literature on the pest. Fecundity for the MN population was numerically highest at 23 °C but was not significantly different from that of the PA population of *H. halys* (212.25 ± 31.04) at 25 °C. In contrast, fecundity of the Oregon, US (78.4 at 22 °C, 141.1 at 25 °C, 76.3 at 27 °C) and Korean (32.3 ± 6.25 at 25 °C, 64.3 ± 10.74 at 30.3 °C, 22.1 ± 4.98 at 32.7 °C) populations were multiple fold (two-nine fold) lower than that of the MN population (Table 2; *p* < 0.05) over the comparable thermal range [46,48]. The fecundity of the Korean population was also negligibly low (8.3 ± 4.05) at 18.8 °C. Such differences in pre-oviposition period and fecundity could be attributed to genetic differences in the geographic populations, differences in the food resources or differences in the date of collection of *H. halys* used in the study [47].

Available evidence to date, suggests that the US populations of *H. halys* trace their origin to three separate primary invasions from China and a secondary invasion within the US, and they are genetically different from each other and the Korean population [74,75,76]. Most relevant, population(s) of *H. halys* in the eastern US (samples from NJ, MD, GA, DE, MA, MS, NY, PA, VA, WV, OH, and MI), states that span a distance of 1287–2253 km, are represented by the same Haplotype H1. In the Northwestern US (samples from OR and WA), populations consisted of 4 haplotypes (H1, H3, H23, H47), but had only a single sample of H1, whereas the Korean population consisted of 8 haplotypes (H2, H22, H25, H28, H29, H35, H36, H37), and is strikingly different [50,74,76,77]. Among the eastern US populations studied [74,75,76], OH and MI have the same H1 haplotype, and are geographically closest (< 1126 km) to the MN population. Thus, it is plausible that our findings for the Minnesota-acclimated population are most indicative of the H1 haplotype of *H. halys.* However, additional haplotype analysis with samples from north-central US populations, including Minnesota, are warranted to fully ascertain their genetic identity [50,77,78]. Irrespectively, our results add new information on temperature-dependent longevity and fecundity of *H. halys*, which is in its early phase of local adaptation and establishment in the MN. The MN climate and photoperiod conditions are distinct relative to much of the pest’s native range in China, and the recent regions of invasion in both the Pacific Northwest and the Atlantic coast states of the US.

Conventionally, the optimal temperature ($T_{opt}$) for poikilothermic organisms is the temperature where maximum developmental rate occurs, and ignores influence on survival and fecundity. For the PA population of *H. halys*, the optimal temperature for development, $T_{opt}$ was assumed to be 25 °C [47]. However, it can be algebraically computed [79] as 30.45 °C, using $T_{0}$ (14.17 °C) and $T_{max}$ (35.76 °C) as input parameters from their best fit Briere-I phenology model. On a similar note, Baek et al. [46], in a comprehensive study, estimated optimal temperatures for peak development of egg (32 °C) and nymphs (30.8 °C), survival rates of egg (24.2 °C) and nymphs (24.5 °C), and fecundity of females (30.1 °C) of the Korean population of *H. halys*. They determined 27.5 to 31.7 °C to be the thermal zone that bracketed the optimal thermal range (B80) (zone with ≥ 80 % performance of maximal rate) for these geographically isolated Korean populations [46], but did not explore beyond. Notably, all of these studies involved a subjective estimate of the optimal temperature for *H. halys* in the midst of conflicting effects of temperature on the different population parameters that render inferences inconclusive [70]. A pertinent question is how we determine the thermal optimum that favors the overall biological success of a population, such as MN-acclimated *H. halys*, that has mean generation time minimized at 30 °C, survival rates enhanced at 27 °C and fecundity maximized at 23 °C?

**Figure 5:**
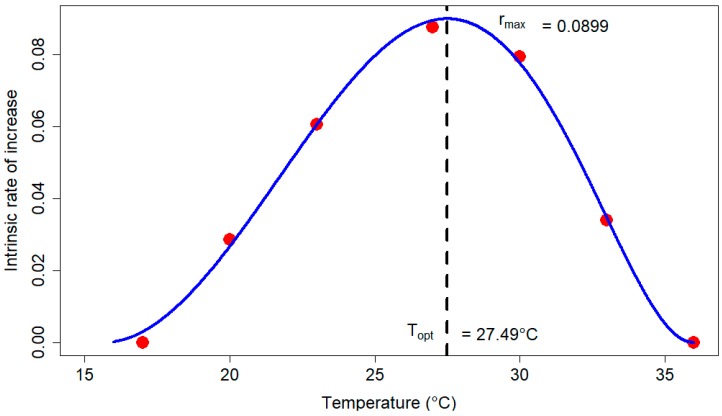
The effect of temperature on the intrinsic rate of increase in *H. halys*, as described by the Ratkowsky model, where the maximum rate of increase is estimated to occur at 27.5 °C.

The intrinsic rate of natural increase ($r_{m}$), is a unique demographic parameter that integrates three different age-stage specific life history parameters of a population, namely developmental rate, survival rate, and reproduction, into one metric [9,70]. As noted earlier, previous research on the effect of temperature on life history parameters of *H. halys* did not estimate $r_{m}$ to compare different populations [10,46,47]. We found that the intrinsic rate of increase was positive between 20 and 33 °C (Table 3), suggesting that the MN population should be productive in this range. Among the tested temperatures, the maximum $r_{m}$ was at 27.5 °C (Figure 5).

Notably, consistent with the maximum $r_{m}$ for the MN population at 27 °C, the life history parameters such as pre-adult development time were rather low (33.21 ± 0.36; not significantly different from the numerically minimum value recorded at 30 °C). Also, maximum fecundity (278.92 ± 57.05; not statistically different from the numerically maximum value recorded at 23 °C) was also recorded at 27 °C. Furthermore, mean generation time was also considerably low, and the net reproduction rate (151.06 ± 41.16), finite rate of increase (1.092 ± 0.00) and survival rates (0.96 ± 0.04) were the maximum at 27.5 °C (Figure 5). Collectively, the lowest development (or generation) time, highest survival, and highest fecundity rates were recorded at 27 °C. Importantly, this meant that an MN population of *H. halys* in stable age-stage distribution and constrained only by temperature could increase at 27 °C by roughly 1.092 times per day (exponential rate of increase of 0.0876 day^−1^) with an average generation time of approximately 56.83 days. The Ratkowsky model [80] provided the best fit to explain the temperature-dependency of $r_{m}$ for the MN population (Figure 5). The model predicted $r_{m}$ for the MN population was maximized (Figure 5; 0.0899 day^−1^) at the thermal optimum of 27.49 °C. If the ability to estimate parameters of biological significance is also accounted as a model selection criteria, the best fit model was Sharpe–Schoolfield–Ikemoto model (Supplementary Figure S1) with the thermal optimum (maximum $r_{m}$ of 0.0983 day^−1^) registered at 28.03 °C. For the OR population, the observed maximum $r_{m}$ (0.027 day^−1^) at 27 and 30 °C [48] was three fold lower relative to that for the MN population. But validity of their estimate is questionable (See, Introduction) and do not deserve merit for further discussion.

While the MN and PA populations of *H. halys* were in agreement regarding overall temperature dependency of life history parameters, the two populations differed for the most part in development time; i.e., for eggs, nymphal and pre-adult phase combined (Table 1, Supplementary Table S3), and the cohort specific survival rates (Table 3) at each temperature. Net reproduction rate, $R_{0}$ (60.02) for the PA population at 25 °C is apparently lower relative to the best corresponding comparable estimate for the MN population at 27 °C ($R_{0}$ = 151.06 ± 41.16). By contrast, however, the corresponding comparisons of the mean generation time, T (56.59) and intrinsic rate of increase, $r_{m}$ (0.07) for the PA and MN (T = 56.83 ± 1.93; $r_{m}$ = 0.0876 ± 0.00) populations are quite similar and suggest only slight differences in these key life history parameters.

An immediate question then is, do observed differences in developmental time and other life history features between the MN and PA populations also suggest that these two populations are genetically distinct? For insect species with continental distributions like *H. halys*, local populations can be considerably different in genetics, physiology, and behavior [68]. Nevertheless, as stated earlier, we considered the MN population to be representative of the eastern US populations [77] and hence share the same haplotype as that of the PA population in the US, along with the Emilia-Romagna population in Italy and the Beijing population in China, all of which are H1 haplotypes [76,81]. For the latter two populations, life history data on developmental time under ambient conditions to compare against the MN population were not available. Albeit, the significantly shorter development time and higher survival for the MN population of *H. halys* reared under comparable conditions in the laboratory (27 °C, 16:8 L:D, 70 ± 10% RH) relative to the PA population (Supplementary Table S1, Table S3 and Table 1) contradicted our expectations of their life history parameters. However, two populations that are not genetically distinct can also have variable life history parameters in the laboratory owing to differences in diet [72]. Consistent with our estimates for the MN population at 27 °C (33.21 ± 0.36 days; 96% survival), a North Carolina population of *H. halys* reared on a mixed food substrate was shown to develop significantly faster (33.9 ± 1.9 days) and exhibit higher survival (85.4%) [72] compared to the PA population (35.8 ± 0.5 days; 52.5% survival); all three populations were reared under the same laboratory conditions (27 °C, 16:8 L:D, 70 ± 10% RH). While green beans and Spanish peanut served as food for *H. halys* in the PA study [47], we used green beans, carrot, and soybeans as food [51], which may have led to differences in the life history estimates of the two populations.

Our estimates of *H. halys* development time, survival, fecundity, and adult longevity for the MN local population adds breadth to the existing life history information on this species that is relevant to IPM. Specifically, our estimates provide a foundation for the development of degree-day models as well as more detailed mechanistic phenology and population models for the MN population that can be used to forecast population growth rates and population dynamics. Specifically, population growth can be studied using various climate change scenarios relevant to the Midwest U.S. region, or in response to IPM strategies. Future research must also direct additional effort to validate temperature-dependent models for development, reproduction, and survival of *H. halys* population in the field, in response to fluctuating temperatures. The phenological model developed [49] for this invasive pest should be strengthened with new information on temperature-dependent fecundity and longevity, and coupled to a phenological model built for the major crops that are largely threatened by this invasive pest. Coupling crop model and pest models enable IPM Extension staff, farm managers and crop consultants to design and implement economical and ecologically viable strategies to manage invasive pest species, with reduced environmental impacts. Our study also provides a strong foundation for further fundamental studies on pest and natural enemy relationships of *H. halys,* including its native parasitoid *Trissolcus japonicus* [82]. Development of crop-pest-parasitoid mechanistic models [83] will be useful to test the implications of various biotic and abiotic factors on tri-trophic dynamics and accompanied variability in biocontrol and crop yield losses in a warming climate for advancing IPM of this invasive insect pest.

Global change, with a projected average warming of 1.5–2 °C (low emission scenario) to 5–6 °C (high emission scenario) by 2100, compared to pre-industrial levels for Minnesota [84], is likely to advance the overall biological fitness of *H. halys* at the current limit of its northern range in North America. The 30-year historical (1981–2010) average monthly summer temperatures from June to August for the Midwest, including the Twin Cities region (MN, USA) range from 22 to 23 °C, and the corresponding average maximum temperatures range from 26 to 28 °C. The prevailing weather conditions are thus ideal to enhance rates of survival, development, and reproduction of the pest at its current northern range limit; additional, projected warming will exacerbate the favorable conditions. Recent significant increases in trap catch numbers of *H. halys* in the seven-county metropolitan region of the Twin Cities [52] suggests the role of an “urban heat island” effect that may facilitate the continued invasion and spread of a pest such as *H. halys,* particularly where urban structures are in ample supply to provide added shelter during winter. Moreover, increases in average diurnal and nocturnal temperatures during summer and winter, respectively [84], are also expected to play a key role in reducing development time, and increase survival rates of *H. halys* in the Minnesota and Midwest region [42]. Considering all aspects of our findings, *H. halys* is thus likely to not only persist in Minnesota, but also build populations and expand its range throughout newer regions where both suitable temperature regimes and habitat exist [85]. Additional research is needed to further assess to what extent *H. halys* will emerge as an economic pest of horticultural and agronomic crops. 

## 5. Conclusions

The present study investigated the influence of temperature on developmental, survival and reproductive rates, longevity and population growth potential of the invasive *H. halys* in Minnesota. Age-stage, two-sex life table analysis showed that the Minnesota population developed faster and survived at higher rates relative to a Pennsylvania population. We used the intrinsic rate of increase, the life table parameter that integrates the effects of temperature on development, survival, and reproduction to determine the thermal optimum favoring overall biological success of a population. For the Minnesota population of *H. halys*, we report the corresponding thermal optimum to be 27.5 °C. Our findings suggest that the projected climate warming scenarios for Minnesota and the Midwest U.S. region are likely to advance the overall biological fitness of the pest, help build populations, allow for continued expansion of its range into new areas, and likely become a growing concern for several summer-autumn crops in the region. Our estimates of life history parameters for *H. halys* can be coupled with developmental rate and degree-day models, to contribute to more detailed mechanistic phenology and population models. This analysis will allow for an improved understanding of the response of pest dynamics to climate change, as well as the development of improved integrated pest management strategies for *H. halys* in the Midwest U.S. region.

## Figures and Tables

**Table 1:** Mean developmental time (days ± standard error [s. e.]) and egg-to-adult survival (%) for MN-acclimated *Halyomorpha halys* at constant temperatures.

Temperature (°C)	n	Egg	First Instar	Second Instar	Third Instar	Fourth Instar	Fifth Instar	Pre-adult	Survival (%)
15	59	21.01± 0.07 ^a^	26.57 ± 0.18 ^a^	—	—	—	—	—	0.00
17	50	13.23 ± 0.04 ^b^	13.20 ± 0.14 ^b^	24.95 ± 0.80 ^a^	22.16 ± 1.55 ^a^	21.43 ± 2.18 ^a^	19.50 ± 0.66 ^a^	105.89 ± 4.69 ^a^	18.00
20	35	12.71 ± 0.11 ^c^	10.90 ± 0.10 ^c^	18.77 ± 0.37 ^b^	12.56 ± 0.90 ^b^	11.15 ± 0.49 ^b^	16.46 ± 0.45 ^a^	80.67 ± 1.04 ^b^	68.00
23	51	5.61 ± 0.09 ^d^	4.95 ± 0.06 ^d^	11.13 ± 0.29 ^c^	8.39 ± 0.30 ^c^	7.90 ± 0.38 ^c^	11.47 ± 0.42 ^b,e^	49.07 ± 1.06 ^c^	82.00
25	51	5.60 ± 0.07 ^d^	5.06 ± 0.10 ^d^	9.58 ± 0.18 ^d^	7.07 ± 0.17 ^c,d^	6.52 ± 0.11 ^c,d^	9.06 ± 0.08 ^c^	42.62 ± 0.38 ^d^	86.00
27	50	3.25 ± 0.04 ^e^	3.80 ± 0.04 ^e^	7.98 ± 0.15 ^e^	5.41 ± 0.11 ^d^	5.38 ± 0.11 ^d^	7.46 ± 0.12 ^d^	33.21 ± 0.36 ^e^	96.00
30	50	3.00 ± 0.00 ^e^	3.02 ± 0.07 ^f^	6.60 ± 0.19 ^f^	5.41 ± 0.14 ^d^	5.31 ± 0.13 ^d^	8.01 ± 0.25 ^c,d^	30.95 ± 0.45 ^e^	80.00
33	56	3.76 ± 0.10 ^f^	3.25 ± 0.04 ^f^	5.43 ± 0.09 ^g^	6.04 ± 0.22 ^d,e^	6.74 ± 0.53 ^c,d^	8.20 ± 0.25 ^c,d^	31.20 ± 0.56 ^e^	10.00
36	133	3.14 ± 0.03 ^e^	3.27 ± 0.05 ^f^	6.63 ± 0.12 ^f^	7.55 ± 0.31 ^c,e^	5.83 ± 0.28 ^c,d^	8.75 ± 0.18 ^c,d,e^	32.17 ± 0.63 ^e^	2.00

Data are the mean ± standard error. Means in a column followed by different letters are significantly different (Tukey HSD, *p* < 0.05).

**Table 2:** Temperature-dependent adult longevity, pre-oviposition period and fecundity for *H. halys*.

Temperature (°C)	Sample Size (Mating Pairs)	Female Adult Longevity (days)	Male Adult Longevity (days)	Pre-oviposition Period (days)	Oviposition Period (days)	Fecundity (eggs/female life span)
17 *	4	227.88 ± 8.27 ^a^	211.4 ± 8.89 ^a^	-	-	-
20	10	181.5 ± 15.39 ^b^	180.75 ± 6.69 ^b^	32.67± 1.37 ^a^	70.13 ± 12.33 ^a^	199.8 ± 49.57 ^a,c^
23	12	130.08 ± 7.62 ^c^	119.12 ± 8.7 ^c^	14.67 ± 1.09 ^b^	59.25 ± 5.33 ^a^	308.67 ± 30.58 ^a^
27	13	85.96 ± 9.72 ^d^	61.20 ± 4.69 ^d^	11.54 ± 0.76 ^c^	38.71 ± 8.37 ^a,b^	278.92 ± 57.05 ^a^
30	19	84.68 ± 6.00 ^d^	83.62 ± 5.35 ^e^	10.84 ± 0.74 ^c^	26.25 ± 4.70 ^b^	152.84 ± 15.98 ^b,c^
33	3	90.67 ± 13.15 ^d^	89.83 ± 9.81 ^c,d,e^	11.00 ± 0.00 ^c^	24.17 ± 11.46 ^a,b^	121.00 ± 33.08 ^b,c^

Data are the mean ± standard error. Means followed by different letters in the same column are significantly different at 5% level by using paired bootstrap test based on the confidence interval of difference. Standard errors were estimated using 10,000 bootstrap random resampling to reduce the variability of the estimates. * Females did not reproduce at 17 °C, and the egg-to-adult survival at 36 °C was too low to estimate the respective parameters using the age-stage, two-sex analysis software at the corresponding temperatures.

**Table 3:** Population parameters (T, mean generation time [d]; R0, net reproduction rate [offspring/individual]; $r_{m}$, intrinsic rate of natural increase [d-1]; λ, finite rate of increase [d-1]; GRR, gross reproduction rate [offspring/individual] and $l_{a}$, pre-adult survival rate [%]) of H. halys (mean ± SE) as affected by temperature (°C).

Temperature (°C)	*n*	*T*	*R*_0_	$r_{m}$	λ	GRR	*l*_a_
20	29	145.60 ± 5.89 ^a^	68.82 ± 23.96 ^a,b^	0.0286 ± 0.00 ^a^	1.029 ± 0.00 ^a^	229.4 ± 84.03 ^a,b,c,d^	0.6896 ± 0.09 ^a^
23	29	79.60 ± 1.95 ^b^	127.73 ± 30.77 ^b^	0.0606 ± 0.00 ^b^	1.062 ± 0.00 ^b^	189.24 ± 40.02 ^a^	0.8273 ± 0.07 ^b,c^
27	24	56.83 ± 1.93 ^c^	151.06 ± 41.16 ^b^	0.0876 ± 0.00 ^c^	1.092 ± 0.00 ^c^	401.65 ± 55.57 ^b^	0.9582 ± 0.04 ^b^
30	50	50.94 ± 1.55 ^d^	58.12 ± 12.02 ^a^	0.0794 ± 0.00 ^c^	1.083 ± 0.01 ^c^	98.42 ± 19.98 ^c^	0.7999 ± 0.06 ^c^
33	56	51.20 ± 3.74 ^d^	6.80 ± 3.75 ^c^	0.0339 ± 0.01 ^a^	1.035 ± 0.01 ^a^	66.81 ± 31.86 ^d^	0.1096 ± 0.04 ^d^

Data are the mean ± standard error. Means followed by different letters in the same column are significantly different at 5% level by using paired bootstrap test based on the confidence interval of difference. Standard errors were estimated using 10,000 bootstrap random resampling to reduce the variability of the estimates. The ‘n’ denotes bootstrap sample size.

## Supplementary Materials

The following are available online at https://www.mdpi.com/2075-4450/11/2/108/s1. Table S1: Stage-specific apparent survival (%) and the cohort-specific total survival (%), Table S2: Estimated parameters from models fit to assess temperature-dependency of intrinsic rate of increase for *Halyomorpha halys* using the devRate and OPTIMSSI package for R, and the corresponding AIC values for each model, Table S3: Mean developmental time (days) and egg-adult survival (%) for the MN and the PA acclimated *H. halys* at constant temperatures. For the PA population, data is reproduced from Nielsen et al. (2008), Figure S1: Effect of temperature on intrinsic rate of increase of *H. halys*, as described by the Sharpe-Schoolfield-Ikemoto (SSI) model, where maximum rate of increase is estimated to occur at 28.03 °C.
