# Supplementary material for: Influence of Temperature on Age-Stage, Two-Sex Life Tables for a Minnesota-Acclimated Population of the Brown Marmorated Stink Bug (Halyomorpha halys)
Source: Insects. 2020 Feb 7;11(2):108. doi: 10.3390/insects11020108 (PMC7073653; doi:10.3390/insects11020108)
Supplement: Supplementary file 1 [file insects-11-00108-s001.zip › Supplementary File 1.pdf]

Supplementary Material

Influence of Temperature on Age-Stage, Two-Sex Life Tables for a Minnesota-Acclimated Population of the Brown Marmorated Stink Bug, (*Halyomorpha halys*)

Byju N. Govindan, and William. D. Hutchison

Department of Entomology, University of Minnesota, St. Paul, MN 55108, USA

The supplementary material tables (**Table S1**, **S2** and **S3)** and one figure **(Figure S1)** are provided to summarize additional information to support our findings from the research on "Influence of Temperature on Age-Stage, Two-Sex Life Tables for a Minnesota-Acclimated Population of the Brown Marmorated Stink Bug, (*Halyomorpha halys*). **Table S1** presents information on stage specific survival (%) and egg-adult survival (%); the egg-adult survival peaks to 96% at 27 °C and declined towards both lower and upper temperature extremes of 15 °C and 36 °C, respectively. **Table S2** presents estimated parameters from non-linear models fit to data on intrinsic rate of increase for *H. Halys* using the *devRate* [1] and *OPTIMSSI* [2] package for R. The Ratkowsky model [3] with the lowest AIC (-59.52) among all models best fitted the data. **Table S3** is a compilation of mean developmental time (days) and egg-adult survival (%) for the MN and the PA acclimated *H. halys* at constant temperatures. Mean development times for the two populations are compared (α = 0.05) using independent two-sample Student's t-test or Welch's t-test, depending on whether sample variances are equal or unequal, respectively. **Figure S1** presents Sharpe-Schoolfield-Ikemoto (SSI) model fit to data on intrinsic rate of increase for *H. Halys*, and it was the best among all models (AIC= -56.24) when biological significance of parameters is also accounted as a model selection criteria [4].

Supplementary Tables

Table S1. Stage-specific apparent survival (%) and the cohort-specific total survival (%)

Table S2. Estimated parameters from models fit to assess temperature-dependency of intrinsic rate of increase for *Halyomorpha halys* using the *devRate* and *OPTIMSSI* package for R, and the corresponding AIC values for each model

Table S3. Mean developmental time (days ± standard error) and egg-adult survival (%) for the MN and the PA acclimated *H. halys* at constant temperatures. For the PA population, data is adapted from Nielsen *et al.* (2008)

Supplementary Figure

Figure S1. Effect of temperature on intrinsic rate of increase of *H. halys*, as described by the Sharpe-Schoolfield-Ikemoto (SSI) model, where maximum rate of increase is estimated to occur at 28.03 °C

Table S1. Stage-specific apparent survival (%) and the cohort-specific total survival (%)

Temperature (°C)	n	Egg	First Instar	Second Instar	Third Instar	Fourth Instar	Fifth Instar	Cohort-specific Survival
15	59	69.41	96.61	2.00	0.00	-	-	0.00
17	50	100.00	66.00	87.88	75.86	63.64	64.29	18.00
20	35	100.00	96.55	100.00	96.43	88.89	100.00	68.00
23	51	100.00	100.00	100.00	82.76	82.76	82.76	82.00
25	51	100.00	100.00	96.08	98.00	95.83	95.65	86.00
27	50	100.00	100.00	96.00	100.00	100.00	97.92	96.00
30	50	100.00	100.00	96.00	93.75	95.56	93.02	80.00
33	56	91.07	100.00	96.08	71.43	82.86	17.24	10.00
36	133	62.41	55.42	63.04	41.38	41.67	50.00	2.00

Table S2. Estimated parameters from models fit to assess temperature-dependency of intrinsic rate of increase for *Halyomorpha halys* using the *devRate* and *OPTIMSSI* package for R, and the corresponding AIC values for each model

Sl. No.	Model	Parameters	AIC
**devRate Package in R:**			
1	Taylor_81:<br>rT ~ Rm * exp(-1/2 * ((T - Tm)/To)^2)	Rm = 0.0934<br>Tm = 26.9894<br>To = 4.2276	-44.41
2	Lactin_95:<br>rT ~ exp(aa * T) - exp(aa * Tmax - (Tmax - T)/deltaT)	aa =0.1643<br>Tmax = 35.7889<br>deltaT = 6.0816	-29.20
3	Logan6_76:<br>rT ~ phi * (exp(bb * T) - exp(bb * Tmax - (Tmax - T)/deltaT))	phi = 0.0484<br>bb = 0.1665<br>Tmax = 35.7791<br>deltaT = 5.9433	-27.21
**4**	**Ratkowsky_83:**<br>**rT ~ (cc * (T - T1) * (1 - exp(k * (T - T2))))^2**	**cc = 0.0540**<br>**T1 = 15.6621**<br>**T2 = 35.9606**<br>**k = 0.0749**	**-59.52**
5	Beta_16:<br>rT ~ rm * (T2 - T)/(T2 - Tm) * ((T - T1)/(Tm - T1))^((Tm - T1)/(T2 - Tm))	rm = 0.0824<br>T1 = 17.0000<br>T2 =35.8232<br>Tm = 27.8516	-40.45
6	Beta_95:<br>rT ~ exp(mu) * (T - Tb)^aa * (Tc - T)^bb	mu = -13.663<br>Tb =14.424<br>Tc = 36.00<br>aa = 2.833<br>bb = 1.858	-54.04
7	Performance-2:<br>rT ~ cc * (T - T1) * (1 - exp(k * (T - T2)))	cc = 0.0271<br>T1 = 17.2861<br>k = 0.0413<br>T2 = 35.7893	-39.70
8	Root square:<br>rT ~ (bb * (T - Tb))^2	bb = 7.938e-04<br>Tb = -2.299e+02	-21.96
9	Wangengel_98:<br>rT ~ (2 * (T - Tmin)^aa * (Topt - Tmin)^aa - (T - Tmin)^(2 * aa))/((Topt - Tmin)^(2 * aa))	aa = 3.965e-01<br>Tmin = 1.700e+01<br>Topt = 1.356e+05	-20.50
10	Regniere_12:	Tb = 16.4650<br>Tm = 35.9889<br>phi = -0.4248	-50.91

	rT ~ phi * (exp(bb * (T - Tb)) - ((Tm - T)/(Tm - Tb)) * exp(-bb * (T - Tb)/deltab) - ((T - Tb)/(Tm - Tb)) * exp(bb * (Tm - Tb) - (Tm - T)/deltam))	bb = 0.1149<br>deltab = -0.6501<br>deltam = 4.1917	
11	Stinner_74:<br>eqn. 1: rT ~ C/(1 + exp(k1 + k2 * T))<br>eqn. 2: rT ~ C/(1 + exp(k1 + k2 * (2 * Topt - T)))	Did not converge	NA
12	Logan10_76:<br>rT ~ alpha * (1/(1 + cc * exp(-bb * T)) - exp(-((Tmax - T)/deltaT)))	alpha = 0.1981<br>bb = 0.3488<br>cc = 1865.9861<br>Tmax = 35.8791<br>deltaT = 10.6802	-43.68
13	SharpeDeMichele_77:<br>rT ~ ((T + 273.16) * exp((aa - bb/(T + 273.16))/1.987))/(1 + exp((cc - dd/(T + 273.16))/1.987) + exp((ff - gg/(T + 273.16))/1.987))	aa = 37.27<br>bb = 16022.62<br>cc = -855.38<br>dd = -250493.15<br>ff = 436.03<br>gg = 132644.05	-49.12
14	Analytis_77:<br>rT ~ aa * (T - Tmin)^bb * (Tmax - T)^cc	Tmin = 1.073e+01<br>Tmax = 3.600e+01<br>aa = 1.244e-04<br>bb = 1.819e+00<br>cc = 5.584e-01	-27.94
15	Schoolfield_81:<br>rT ~ (p25 * (T + 273.16)/298 * exp(aa/1.987 * (1/298 - 1/(T + 273.16))))/(1 + exp(bb/1.987 * (1/cc - 1/(T + 273.16))) + exp(dd/1.987 * (1/ee - 1/(T + 273.16))))	p25 = 7.391e-02<br>aa = 1.602e+04<br>bb = -2.505e+05<br>cc = 2.928e+02<br>dd = 1.326e+05<br>ee = 3.042e+02	-49.12
16	SchoolfieldHigh_81:<br>rT ~ (p25 * (T + 273.16)/298 * exp(aa/1.987 * (1/298 - 1/(T + 273.16))))/(1 + exp(dd/1.987 * (1/ee - 1/(T + 273.16))))	p25 = 7.501e-02<br>aa = 3.564e+04<br>dd = 1.159e+05<br>ee = 3.023e+02	-37.88
17	SchoolfieldLow_81:<br>rT ~ (p25 * (T + 273.16)/298 * exp(aa/1.987 * (1/298 - 1/(T + 273.16))))/(1 + exp(bb/1.987 * (1/cc - 1/(T + 273.16))))	p25 = 1.208e+00<br>aa = -8.026e+04<br>bb = -1.159e+05<br>cc = 3.023e+02	-37.88
18	Poly2:<br>rT ~ a0 + a1 * T + a2 * T^2	a0 = -0.5850<br>a1 = 0.04983<br>a2 = -0.0009	-40.05
19	HarcourtYee_82:<br>rT ~ a0 + a1 * T + a2 * T^2 + a3 * T^3	a0 = -2.054e-01<br>a1 = 3.799e-03<br>a2 = 8.588e-04<br>a3 = -2.249e-05	-40.16

20	Poly4:<br>rT ~ a0 + a1 * T + a2 * T^2 + a3 * T^3 + a4 * T^4	a0 = 2.973e+00<br>a1 = -5.137e-01<br>a2 = 3.165e-02<br>a3 = -8.167e-04<br>a4 = 7.505e-06	-49.97
21	HilbertLogan_83:<br>rT ~ phi * (((T - Tb)^2/((T - Tb)^2 + aa^2)) - exp(-(Tmax - (T - Tb))/deltaT))	phi = 0.275<br>aa = 10.902<br>Tb = 13.604<br>Tmax = 24.128<br>deltaT = 9.025	-39.33
22	Lamb_92[[1]]:<br>rT ~ Rm * exp(-1/2 * ((T - Tmax)/To)^2)	Rm = 0.0934<br>Tmax = 26.9894<br>T0 = -4.2276	-44.41
23	Lamb_92[[2]]:<br>rT ~ Rm * exp(-1/2 * ((T - Tmax)/To)^2)	Rm = 0.0934<br>Tmax = 26.9894<br>T0 = -4.2276	-44.41
24	Lactin1_95:<br>rT ~ exp(aa * T) - exp(aa * Tmax - (Tmax - T)/deltaT)	aa =0.1643<br>Tmax = 35.7889<br>deltaT = 6.0816	-29.20
25	Lactin2_95:<br>rT ~ exp(aa * T) - exp(aa * Tmax - (Tmax - T)/deltaT) + bb	aa = 0.0288<br>bb = -1.0623<br>Tmax = 51.3935<br>deltaT = 16.8618	-39.35
26	Briere1_99:<br>rT ~ aa * T * (T - Tmin) * (Tmax - T)^(1/2)	aa = 6.991e-05<br>Tmax = 3.600e+01<br>Tmin = 1.544e+01	-29.12
27	Briere2_99:<br>rT ~ aa * T * (T - Tmin) * (Tmax - T)^(1/bb)	aa = 1.892e-05<br>bb = 7.748e-01<br>Tmax = 3.600e+01<br>Tmin = 1.743e+01	-42.66
28	Kontodimas_04:<br>rT ~ aa * (T - Tmin)^2 * (Tmax - T)	aa = 5.208e-05<br>Tmax = 3.568e+01<br>Tmin = 1. 382e+01	-37.85
29	Damos_08:<br>rT ~ aa * (bb - T/10) * (T/10)^cc	aa = -2.351<br>bb = 1.721<br>cc = -3.707	-24.83
30	Damos_11:<br>rT ~ aa/(1 + bb * T + cc * T^2)	aa = 0.0025<br>bb = -0.0716<br>cc = 0.0013	-34.86
31	Wang_82:<br>rT ~ (K/(1 + exp(-r * (T - T0)))) * (1 - exp(-(T - TL)/aa)) * (1 - exp(-(TH - T)/aa))	K =55.1509<br>r = 0.4752<br>aa = 447.4390<br>TL = -20.5821<br>T0 = 23.6922<br>TH = 35.8769	-41.40
32	Bayoh and Lindsay (bayoh_03):	aa = 2.927e-02	-26.11

	rT ~ aa + bb * T + cc * exp(T) + dd * exp(-T)	bb = 1.147e-03<br>cc = -1.670e-17<br>dd = -1.203e+06	
33	Wagner_88<br>rT ~ 1/((1 + exp((cc/1.987) * ((1/dd) - (1/(T + 273.16)))))/(aa * (T + 273.16)/298.15 * exp((bb/1.987) * ((1/298.15) - 1/(T + 273.16)))))	aa = 7.736e-02<br>bb = 3.564e+04<br>cc = 1.159e+05<br>dd = 3.023e+02	-37.88
34	Bieri1_83:<br>rT ~ aa * (T - Tmin) - (bb * exp(T - Tm))	Did not converge	NA
**OPTIMSSI Package in R:**			
35.	Sharpe-Schoolfield-Ikemoto model:<br>$r(T) = \frac{\rho_{\Phi} \frac{T}{T_{\Phi}} \exp\left[\frac{\triangle H_A}{R}\left[\frac{1}{T_{\Phi}} - \frac{1}{T}\right]\right]}{1 + \exp\left[\frac{\triangle H_L}{R}\left[\frac{1}{T_L} - \frac{1}{T}\right]\right] + \exp\left[\frac{\triangle H_H}{R}\left[\frac{1}{T_H} - \frac{1}{T}\right]\right]}$	T_{Φ}= 2.959408e+02<br>ρ_{Φ} = 5.436652e-02<br>$\triangle H_A$ = 2.841494e+04<br>$\triangle H_L$= -2.846197e+05<br>$\triangle H_H$ = 1.241860e+05<br>T_L = 2.925017e+02<br>T_H = 3.029137e+02	-56.24

Note 1: The Ratkowsky non-linear empirical model [3], with the lowest AIC (-59.52) among all models considered [1], best described the relation between temperature and intrinsic rate of increase for *H. halys*. The r_m was maximized (0.0899 day^{-1}) at 27.49˚C.

Note 2: The Sharpe-Schoolfield-Ikemoto (SSI) non-linear biophysical model [2] based on enzyme kinetics approach provided the next best fit (AIC = -56.24). If the ability to estimate parameters of biological significance is also accounted as a model selection criteria [4], the best fit model was SSI model (**Figure S1**). The r_m was maximized (0.0983 day^{-1}) at 28.03˚C.

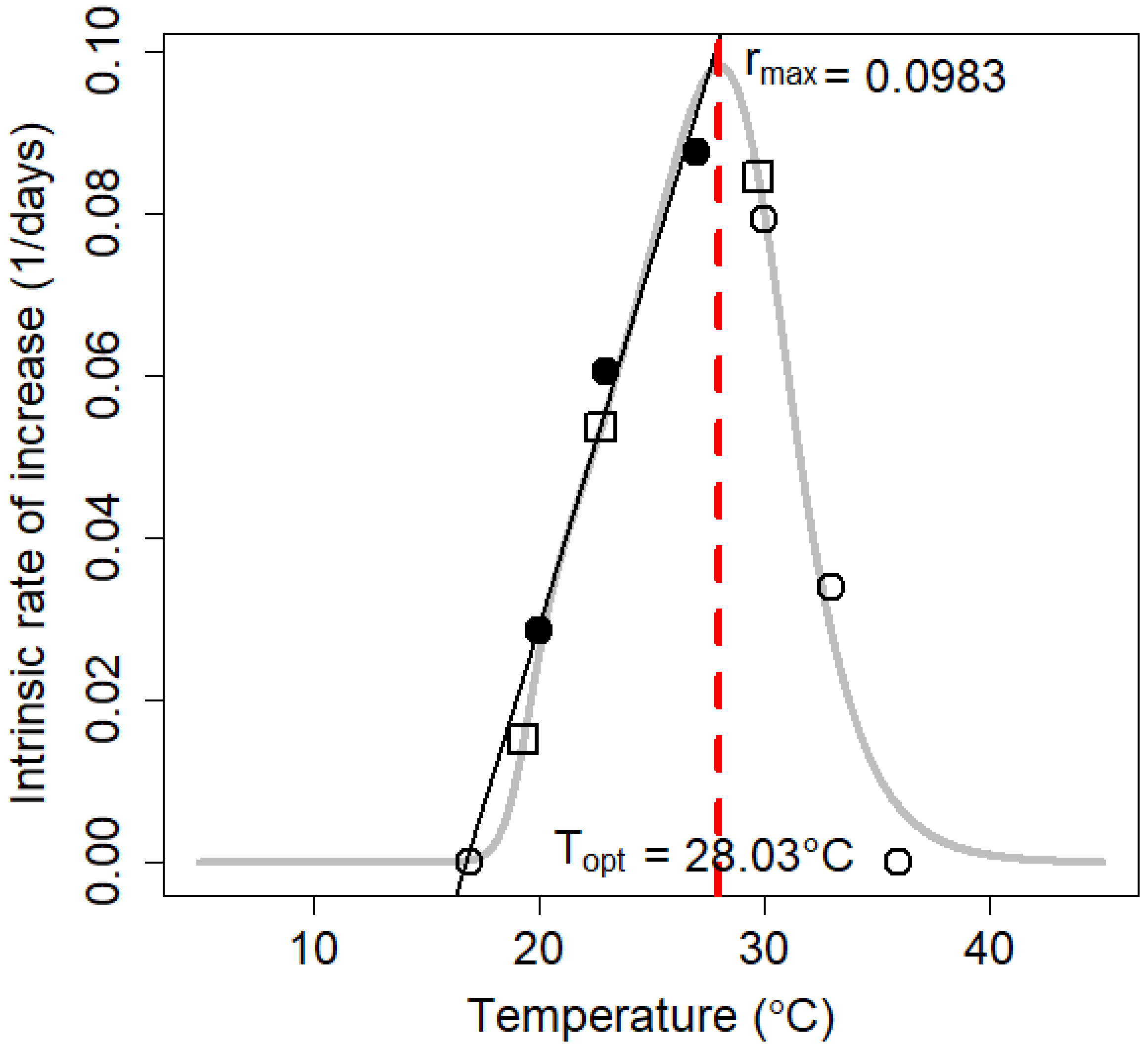

Figure S1. Effect of temperature on intrinsic rate of increase of *H. halys*, as described by the Sharpe-Schoolfield-Ikemoto (SSI) model, where maximum rate of increase is estimated to occur at 28.03 °C

Table S3. Mean developmental time (days ± standard error) and egg-adult survival (%) for the MN and the PA acclimated *H. halys* at constant temperatures. For the PA population, data is adapted from Nielsen *et al.* (2008) [5]

Temperature	n	Egg	First Instar	Second Instar	Third Instar	Fourth Instar	Fifth Instar	Pre-adult	Survival (%)
15°C: MN	59	21.01± 0.07^{a}	26.57 ± 0.18	38.25 ± 1.37	32.59 ± 1.04	28.59 ± 1.23	43.73 ± 1.72	186.23± 0.78	0.00
15°C: PA	50	22.00 ±0.00^{b}<br>(t_{98} = -7.44,<br>P = 3.87 x 10^{-11})	—	—	—	—	—	—	0.00
17°C: MN	50	13.23 ± 0.04^{a}	13.20 ± 0.14^{a}	24.95 ± 0.80^{a}	22.16 ± 1.55	21.43 ± 2.18	17.90 ± 1.63^{a}	103.89 ± 4.69^{a}	18.00
17°C: PA	100	17.20 ± 0.08^{b}<br>(t_{148} = -44.39,<br>P = 2.54 x 10^{-83})	17.01 ± 0.16^{b}<br>(t_{95} = -17.92,<br>P = 1.25 x 10^{-31})	30.36 ± 1.53^{b}<br>(t_{38} = -3.73,<br>P = 0.006)	22.40 ± 4.02	23.00 ± 1.08	28.00 ± 0.00^{b}<br>(t_{7} = -6.20,<br>P = 0.0002)	121.50 ± 0.50^{b}<br>(t_{9} = -3.73,<br>P = 0.006)	2.00
20°C: MN	35	12.71 ± 0.11^{a}	10.90 ± 0.10^{a}	18.77 ± 0.37^{a}	12.56 ± 0.90	11.15 ± 0.49^{a}	16.46 ± 0.45^{a}	80.67 ± 1.04	68.00
20°C: PA	100	11.50 ± 0.05^{b}<br>(t_{149} = -7.79,<br>P = 3.83 x 10^{-9})	9.34 ± 0.08^{b}<br>(t_{118} = 12.03,<br>P = 2.27 x 10^{-18})	16.25 ± 0.23^{b}<br>(t_{102} = 5.78,<br>P = 4.95 x 10^{-7})	11.78 ± 0.29	13.66 ± 0.31^{b}<br>(t_{95} = -4.33,<br>P = 8.82 x 10^{-5})	20.16 ± 0.36^{b}<br>(t_{83} = -6.42,<br>P = 4.29 x 10^{-8})	81.16 ± 0.80	62.00
25°C: MN	51	5.60 ± 0.07^{a}	5.06 ± 0.10	9.58 ± 0.18	7.07 ± 0.17	6.52 ± 0.11^{a}	9.06 ± 0.08^{a}	42.62 ± 0.38^{a}	86.00
25°C: PA	100	6.10 ± 0.03^{b}<br>(t_{149} = -6.57,<br>P = 1.08 x 10^{-9})	4.82 ± 0.10	9.62 ± 0.21	7.08 ± 0.22	7.38 ± 0.28^{b}<br>(t_{107} = -2.86,<br>P = 0.005)	10.44 ± 0.28^{b}<br>(t_{103} = -4.74,<br>P = 1.10 x 10^{-5})	44.92 ± 0.80^{b}<br>(t_{103} = -2.60,<br>P = 0.01)	61.00
27°C: MN	50	3.25 ± 0.04^{a}	3.80 ± 0.04^{a}	7.98 ± 0.15	5.41 ± 0.11	5.38 ± 0.11^{a}	7.46 ± 0.12	33.21 ± 0.36^{a}	94.00
27°C: PA	80	4.87 ± 0.10^{b}<br>(t_{128} = -15.04,<br>P = 1.20 x 10^{-27})	4.25 ± 0.05^{b}<br>(t_{128} = -7.03,<br>P = 1.11 x 10^{-10})	7.64 ± 0.19	5.49 ± 0.21	5.90 ± 0.18^{b}<br>(t_{97} = -2.47,<br>P = 0.02)	7.81 ± 0.28	35.81 ± 0.52^{b}<br>(t_{87} = -4.11,<br>P = 0.0001)	52.50
30°C: MN	50	3.00 ± 0.00	3.02 ± 0.07^{a}	6.60 ± 0.19^{a}	5.41 ± 0.14^{a}	5.31 ± 0.13^{a}	8.01 ± 0.25	30.95 ± 0.45^{a}	80.00
30°C: PA	100	3.00 ± 0.00	3.70 ± 0.05^{b}<br>(t_{142} = -7.03,<br>P = 8.29 x 10^{-11})	7.05 ± 0.13^{b}<br>(t_{128} = -2.01,<br>P = 0.05)	6.11 ± 0.28^{b}<br>(t_{117} = -2.23,<br>P = 0.03)	6.11 ± 0.23^{b}<br>(t_{103} = -3.14,<br>P = 0.002)	8.47 ± 0.28	33.39 ± 0.50^{b}<br>(t_{89} = -3.63,<br>P = 0.0005)	51.00
33°C: MN	56	3.76 ± 0.10^{a}	3.25 ± 0.04^{a}	5.43 ± 0.09^{a}	6.04 ± 0.22^{a}	6.74 ± 0.53	8.20 ± 0.25^{a}	31.20 ± 0.56^{a}	10.00
33°C: PA	95	4.00 ± 0.00^{b}<br>(t_{149} = -2.40,<br>P = 0.02)	3.01 ± 0.01^{b}<br>(t_{106} = -5.10,<br>P = 4.51 x 10^{-6})	7.47 ± 0.23^{b}<br>(t_{76} = -8.26,<br>P = 6.77 x 10^{-10})	7.45 ± 0.53^{b}<br>(t_{53} = -2.46,<br>P = 0.02)	7.20 ± 0.40	10.60 ± 0.81^{b}<br>(t_{8} = -2.83,<br>P = 0.04)	37.80 ± 0.86^{b}<br>(t_{8} = -6.43,<br>P = 0.0004)	5.00

Data are mean ± standard error. Estimates for the PA population of BMSB are from Nielsen *et al.* 2008). The 'n' denotes sample size (# of eggs) at the beginning of the experiment. For each instar (or phase) at a given temperature, the means in a column followed by a different letter suggest that estimates for the MN and for the PA population of *Halyomorpha halys* are significantly different (t test, $P < 0.05$). Comparisons were not possible for 23°C and 36°C, due to lack of estimates for the PA BMSB. An em dash [—] suggests lack of data owing to 100% mortality.

References:

1. Rebaudo, F.; Struelens, Q.; Dangles, O. Modelling temperature-dependent development rate and phenology in arthropods: The devRate package for r. *Methods Ecol. Evol.* **2018**, *9*, 1144–1150.
2. Shi, P.; Ikemoto, T.; Egami, C.; Sun, Y.; Ge, F. A modified program for estimating the parameters of the SSI model. *Environ. Entomol.* **2011**, *40*, 462–469.
3. Ratkowsky, D.A.; Olley, J.; McMeekin, T.A.; Ball, A. Relationship between temperature and growth rate of bacterial cultures. *J. Bacteriol.* **1982**, *149*, 1–5.
4. Bahar, M.H.; Soroka, J. J.; Grenkow, L.; Dosdall, L. M. New threshold temperatures for the development of a North American diamondback moth (Lepidoptera: Plutellidae) population and its larval parasitoid, *Diadegma insulare* (Hymenoptera: Ichneumonidae). *Environ Entomol*. **2014**, *43*, 1443-1452.
5. Nielsen, A.L.; Hamilton, G.C.; Matadha, D. Developmental rate estimation and life table analysis for *Halyomorpha halys* (Hemiptera: Pentatomidae). *Environ. Entomol.* **2008**, *37*, 348–355.
