# Supplementary figures and images for: Influence of Temperature on Age-Stage, Two-Sex Life Tables for a Minnesota-Acclimated Population of the Brown Marmorated Stink Bug (Halyomorpha halys)
Source: Insects. 2020 Feb 7;11(2):108. doi: 10.3390/insects11020108 (PMC7073653; doi:10.3390/insects11020108)

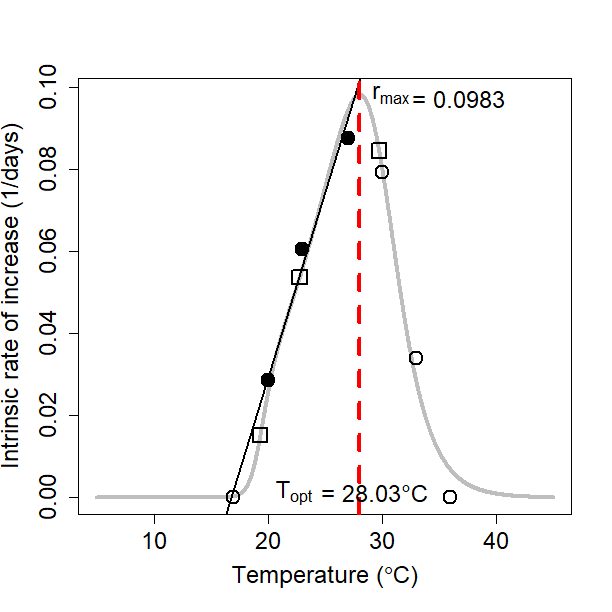

Supplement: Supplementary file 1 [file insects-11-00108-s001.zip › Supplement Fig S1/Suppl. Fig S1. IntrinsicRate(BMSB)VsTemp.tiff]
